# Awareness and attitudes toward passive smoking: sociodemographic correlates and public health implications from a cross-sectional study in Saudi Arabia

**DOI:** 10.3389/fpubh.2025.1683306

**Published:** 2025-10-17

**Authors:** Azharuddin Sajid Syed Khaja, Hamad Rudhayman Alrashdi, Osama Abdullah Almuzaini, Hatim Salem Alanzi, Rabah Abdulaziz Alshuhail, Khursheed Muzammil, Sheeba Afreen, Mohd Saleem

**Affiliations:** ^1^Department of Pathology, College of Medicine, University of Hail, Hail, Saudi Arabia; ^2^Medical Student, College of Medicine, University of Hail, Hail, Saudi Arabia; ^3^Central Labs, King Khalid University, AlQura'a, Abha, Saudi Arabia; ^4^Department of Public Health, College of Applied Medical Sciences, King Khalid University, Abha, Saudi Arabia; ^5^Department of Biochemistry, Career Institute of Medical Science and Hospital, Lucknow, India

**Keywords:** passive smoking, secondhand smoke exposure, awareness and attitudes, public health, sociodemographic factors, principal component analysis (PCA)

## Abstract

**Background and Aim:**

Passive smoking, or secondhand smoke exposure, poses a major public health risk linked to various adverse outcomes. This study aimed to assess sociodemographic characteristics, exposure patterns, and levels of awareness and attitudes toward passive smoking among a group of volunteers in Saudi Arabia.

**Methods:**

A descriptive cross-sectional design was applied, with 301 participants enrolled through convenience sampling. The sample included members of the public and university students, including medical students, to enable educational-level comparisons. Individuals unwilling to participate, unable to provide consent, or unable to complete the survey were excluded. Data were obtained via a structured, pilot-tested questionnaire that assessed demographics, SHS exposure, knowledge, and attitudes. Reliability was confirmed (Cronbach's α = 0.78), and principal component analysis (PCA) was used to extract key awareness and attitude dimensions.

**Results:**

The study cohort was predominantly aged 18–30 years (65.1%), held college degrees (76.4%), and resided in urban areas (84.4%). While 84.1% were non-smokers, over one-third lived with smokers, and 42.5% reported weekly exposure, most often in public venues. Awareness of SHS risks was high: 94.4% recognized its harmful effects, and 89.0% identified children and pregnant women as particularly vulnerable. Support for smoke-free policies was also strong (85.7%). The PCA identified four components: general knowledge, advocacy, tolerance of smoking behavior, and perceptions of policy. Overall, 85.7% demonstrated good awareness and attitudes.

**Conclusion:**

This study highlights high awareness and strong support for smoke-free policies among participants. These findings support the need for continued education and stronger regulatory efforts to minimize passive smoke exposure, particularly in public areas.

## 1 Introduction

Tobacco use has long been recognized as one of the leading causes of preventable morbidity and mortality worldwide, accounting for more than eight million deaths annually (1–3). While the adverse health effects of active smoking are well established, exposure to secondhand smoke (SHS), also referred to as passive smoking, remains an equally pressing public health concern. SHS refers to the involuntary inhalation of tobacco smoke from the surrounding environment, which often exposes non-smokers without their consent (1, 2, 4). The World Health Organization (WHO) emphasizes that there is no safe level of SHS exposure and identifies it as a major public health hazard associated with cardiovascular diseases, respiratory illnesses, and various cancers, with children and non-smoking adults being particularly vulnerable (2, 5).

Globally, exposure to SHS remains a major public health concern. Data from the Global Youth Tobacco Survey (2010–2018) across 142 countries indicated that approximately 62.9% of adolescents reported SHS exposure in any place for at least one day during the past week, with 33.1% exposed at home and 57.6% in public places (6). In Saudi Arabia, the prevalence of SHS exposure among adolescents remains high. National survey data reported that 32.3% of adolescents were exposed to SHS at home. In comparison, 40.8% reported exposure in public places, underscoring the persistence of SHS exposure in both private and public environments despite tobacco control measures (7). In addition to its well-documented physical health consequences, including increased risks of ischemic heart disease, chronic obstructive pulmonary disease, stroke, and lung cancer (3), SHS is also linked to adverse psychological outcomes, highlighting the need to view its impact in a broader health context. Evidence from Spain and Canada indicates that domestic SHS exposure can negatively impact mental health, potentially through biobehavioral mechanisms such as stress pathway activation and neurochemical disruption (5, 8, 9). Individuals with pre-existing mental health conditions may face heightened vulnerability due to nicotine-induced dysregulation of dopaminergic pathways (10).

Despite these well-documented health risks, a significant gap often exists between awareness of SHS dangers and the adoption of protective behaviors, which are heavily influenced by sociocultural and environmental contexts. In many regions, such as South Asia, smoking is often normalized in homes and public spaces, making exposure a significant concern (11). Studies from Nepal and India revealed considerable knowledge gaps even among medical students and adolescents, highlighting insufficient awareness and weak behavioral responses to SHS (12, 13). Adolescents, in particular, are at high risk because exposure commonly occurs at home or in social settings, often without an adequate understanding of its harmful effects (14). Misconceptions about SHS are widespread, as shown in Malaysia, Bangladesh, and Jordan, where low awareness correlates with higher exposure (15–17).

Education appears to play a protective role in reducing SHS exposure. In Jordan, educated non-smoking women are more likely to avoid SHS due to greater awareness of its risks (17), whereas Portuguese university students reported that knowledge and attitudes significantly influence both smoking behavior and SHS exposure (18). However, awareness does not always translate into behavior change. In Bangladesh, many students who understand SHS risks fail to adopt avoidance behaviors (19). Similarly, Afiah et al. (20) reported that undergraduates with stronger attitudes and awareness were more likely to adopt protective behavior. Cultural norms and domestic practices further complicate SHS exposure, especially in South Asian and African contexts (21). In India, households have become primary exposure sites because of indoor smoking by male family members, whereas women report variable levels of prevention knowledge (22). Similarly, in Nigeria, frequent SHS exposure in homes and social gatherings coexists with low awareness among non-smokers (23). Even healthcare students, who are expected to advocate for tobacco control, often display high smoking rates and poor knowledge of SHS, highlighting the need for curriculum reforms and stronger institutional policies (24). Furthermore, SHS exposure is linked to other risky behaviors, especially among adolescents, where peer influences and shared environments create a convergence of risk factors necessitating integrated interventions (25, 26).

Given the global burden of passive smoking, coupled with variable awareness, cultural normalization, and limited policy enforcement, there is a pressing need for evidence-based strategies (27, 28). Although efforts to reduce active smoking have made progress, SHS continues to disproportionately affect non-smokers and vulnerable groups (29–31). Furthermore, the interplay between sociodemographic factors, environmental exposure, and public perception is still insufficiently understood (32).

Within this global context, evidence from Saudi Arabia is notably limited. While regional studies indicate high exposure rates, particularly among youth (7), there is a lack of in-depth research assessing public awareness, attitudes, and exposure patterns following the implementation of Vision 2030 health initiatives and stricter tobacco control laws (33–35). The sociodemographic and cognitive factors shaping the public's understanding of SHS risks and support for protective policies are not well characterized. Therefore, this study aims to assess awareness and attitudes toward passive smoking, along with associated sociodemographic factors and exposure patterns within the Hail region of Saudi Arabia, to inform targeted and effective public health interventions and national tobacco control strategies.

## 2 Methodology

### 2.1 Ethical approval and study design

This cross-sectional, observational study was conducted following ethical approval from the University of Hail Ethics Committee (Reference: H-2024-517). The primary objective was to assess awareness and attitudes toward passive smoking and examine associated sociodemographic determinants, including knowledge of health risks, exposure levels, and perspectives on smoke-free policies. The cross-sectional design enabled the assessment of population-level perceptions and behaviors at a single point in time.

### 2.2 Study population and setting

A total of 350 individuals from diverse socioeconomic, educational, and occupational backgrounds across urban, suburban, and rural areas were approached to participate in the study through both online and in-person channels. While the sample captured a spectrum of socioeconomic, educational, and occupational profiles, its demographic distribution was skewed, with a predominant representation of urban (84.4%) and young adult populations. The participants included members of the general public and university students, including medical students, to allow comparisons by educational background. Data were collected in academic institutions and community venues to capture diverse perspectives. The exclusion criteria included individuals who were unwilling to participate, those unable to provide informed consent due to cognitive impairment, and those unable to complete the questionnaire.

Of the 350 individuals approached, 326 initiated the questionnaire (initial response rate: 93.1%). After 25 incomplete responses were excluded, a final sample of 301 complete and valid responses was retained for analysis, yielding an effective participation rate of 86.0%. The age of the participants ranged from under 18 years to over 60 years, offering a broad demographic spectrum to examine variation in awareness and exposure across age cohorts.

### 2.3 Sample size and sampling technique

The participants were selected via a nonprobability convenience sampling approach. This method was selected on the basis of logistical considerations and the need for timely data collection. Although convenience sampling may introduce bias, participants were recruited through both online and in-person channels in academic institutions and community settings, which allowed the inclusion of individuals from different age groups, educational levels, occupations, and residential areas, thereby enhancing sample diversity despite the absence of formal stratification or quotas.

### 2.4 Data collection tool

Data were collected via a structured questionnaire, which was developed after a comprehensive review of literature on SHS exposure, awareness, and attitudes. The items were adapted from prior surveys (12, 13, 16, 17, 36), translated into Arabic and culturally adapted to ensure relevance and appropriateness for the Saudi Arabian context. Content validity was ensured through expert review by faculty members in public health and epidemiology. A pilot test was conducted on a small group of participants (n = 20) to refine clarity, language, and relevance. Reliability testing via Cronbach's alpha demonstrated acceptable internal consistency (α = 0.78).

The final questionnaire was structured into four main sections. The first section collected sociodemographic data, including age, gender, education, occupation, income, and residence. The second section focused on SHS exposure, capturing details on participants' personal smoking status, the frequency and duration of their exposure, and common exposure sites. The third section assessed awareness and attitudes through 15 items related to perceived health risks, vulnerable populations, and support for tobacco control measures. The fourth section contained variables for statistical analysis, with awareness and attitude items further analyzed via principal component analysis (PCA).

### 2.5 Data collection procedure

The questionnaire was administered in both online and paper-based formats to ensure accessibility and inclusiveness. Online distribution utilized email and social media platforms, while paper copies were disseminated in educational and community venues. Trained research assistants, who underwent structured training on study objectives, standardized administration, and the handling of participant queries, facilitated the process and ensured completeness. Their work was closely supervised by the principal investigator to maintain consistency and adherence to the protocol. As the questionnaire was primarily self-administered, the scope for inter-rater variability was limited. Informed consent was obtained from all participants, and confidentiality was maintained throughout. Data collection was completed within 4 weeks.

### 2.6 Statistical analysis

The data were analyzed via IBM SPSS Statistics software (version 23). Descriptive statistics, including frequencies and percentages, were computed for sociodemographic characteristics, exposure levels, and awareness/attitude responses. PCA was conducted to identify latent constructs within the awareness and attitude items, with components extracted on the basis of the Kaiser criterion (eigenvalues >1) and subjected to varimax rotation. Chi-square tests were used to examine associations between sociodemographic variables and categorized levels of awareness and attitudes. Statistical significance was defined as a *p*-value < 0.05.

## 3 Results

### 3.1 Sociodemographic characteristics of the participants

**Figure 1:**
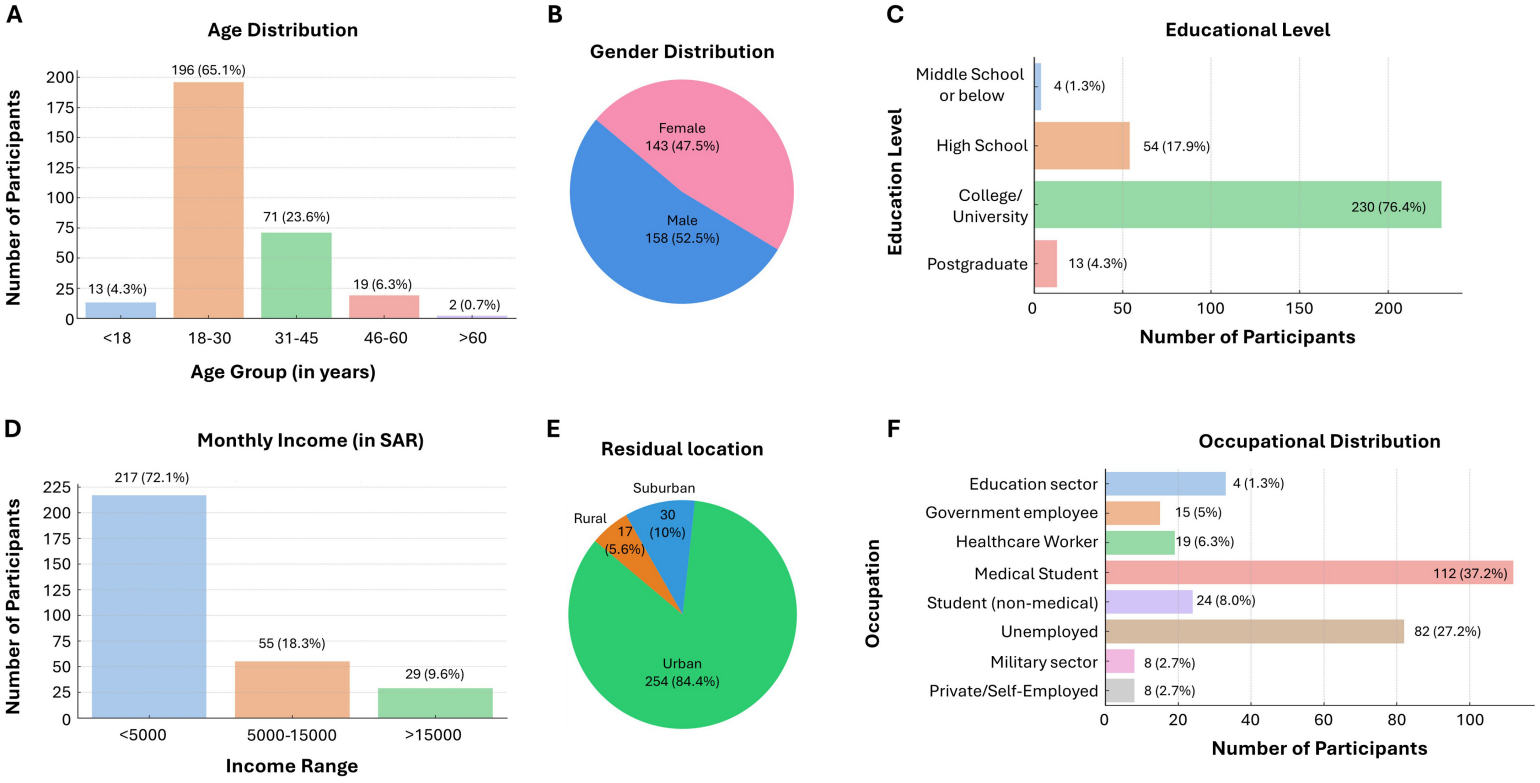
Sociodemographic characteristics of the participants. **(A)** Age distribution, with most participants aged 18–30 years (65.1%). **(B)** Gender distribution showing 52.5% males and 47.5% females. **(C)** Educational levels, with 76.4% holding a college or university degree. **(D)** Monthly income, with 72.1% earning less than 5,000 Saudi Riyals (SAR). **(E)** Residential location, primarily urban (84.4%). **(F)** Occupational breakdown, led by medical students (37.2%) and unemployed individuals (27.2%).

The sociodemographic profile of the participants revealed that the majority were between 18 and 30 years of age (65.1%), followed by those aged 31–45 years (23.6%), with smaller proportions in the < 18 (4.3%), 46–60 (6.3%), and >60 years (0.7%) age brackets (Figure 1A). In terms of gender (Figure 1B), there was a slightly greater percentage of males (52.5%) than females (47.5%). A large majority had attained a college or university degree (76.4%), with 17.9% completing high school, 4.3% holding a postgraduate qualification, and only 1.3% educated up to middle school or below (Figure 1C).

In terms of monthly income, the majority (72.1%) earned less than 5,000 Saudi Riyals (SAR), 18.3% earned between 5,000 SAR and 15000 SAR, and only 9.6% had a monthly income exceeding 15,000 SAR (Figure 1D). Most participants resided in urban areas (84.4%), with smaller groups in suburban (10.0%) and rural (5.6%) regions (Figure 1E). With respect to occupation (Figure 1F), 37.2% were medical students, whereas 27.2% were unemployed. Others were from the education sector (11.0%), healthcare (6.3%), government jobs (5.0%), non-medical students (8.0%), the military sector (2.7%), and private/self-employment (2.7%).

### 3.2 Smoking status and exposure to passive smoking

**Figure 2:**
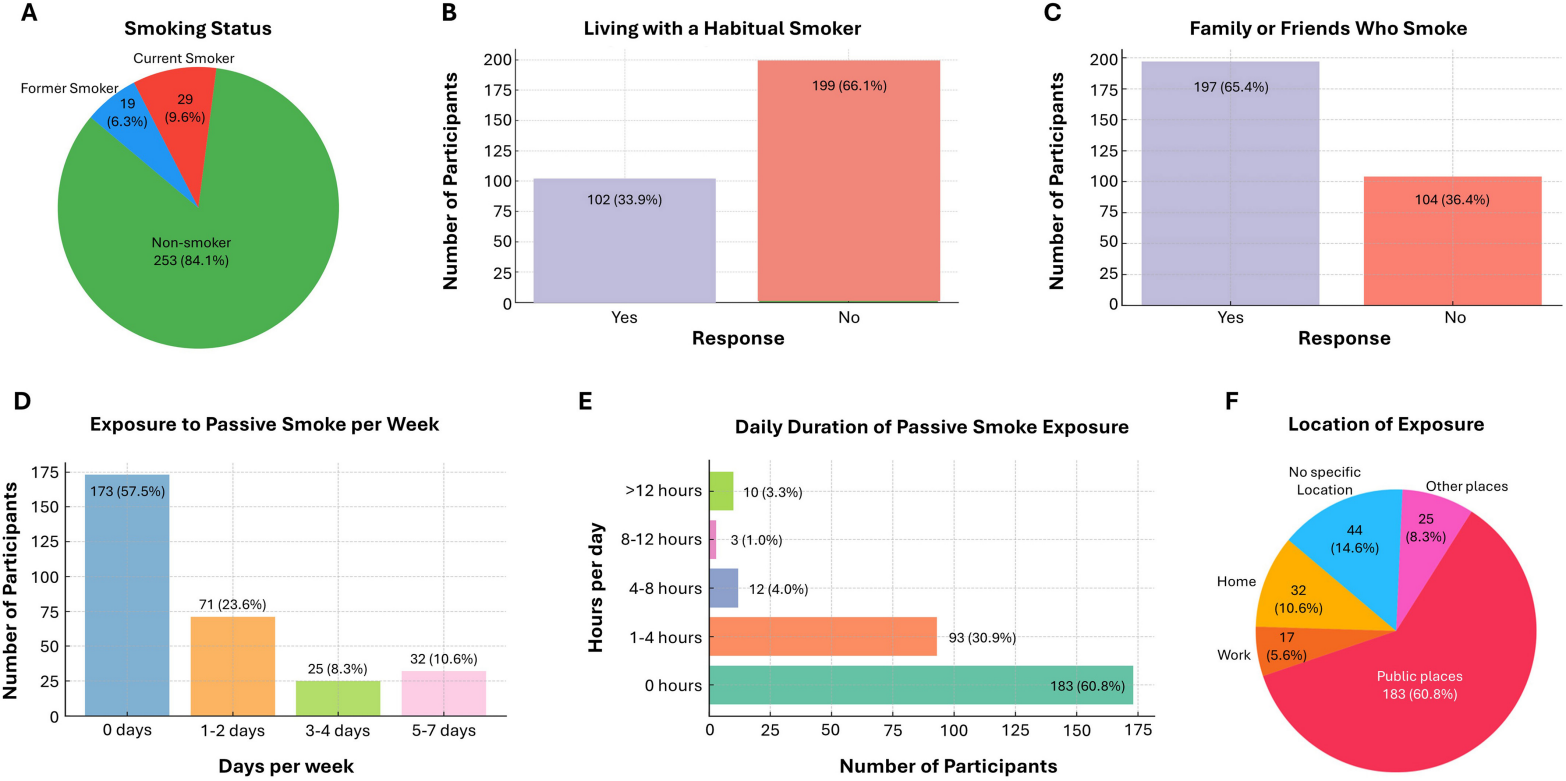
Exposure to Smoking and Secondhand Smoke Among the Participants **(A)** Distribution of participants according to smoking status; **(B)** proportion of participants living with a habitual smoker; **(C)** presence of smokers among family or friends; **(D)** frequency of weekly passive smoke exposure; **(E)** daily duration of passive smoke exposure; **(F)** main settings where passive smoke exposure occurs.

The data on exposure to smoking revealed that a majority of participants were non-smokers (84.1%), whereas 9.6% identified as current smokers and 6.3% as former smokers (Figure 2A). About one-third (33.9%) reported living with a habitual smoker, and nearly two-thirds (65.4%) had family members or friends who smoked (Figures 2B, C). In terms of passive smoke exposure, 57.5% reported no exposure, while 23.6% were exposed 1–2 days per week, 8.3% for 3–4 days, and 10.6% for 5–7 days (Figure 2D). With respect to daily exposure duration, 60.8% were not exposed, but 30.9% were exposed for 1–4 hours daily, 4.0% for 4–8 hours, 1.0% for 9–12 h, and 3.3% for more than 12 h (Figure 2E). Public places were the most common location of exposure (60.8%), followed by home (10.6%), other settings (8.3%), and work (5.6%), while 14.6% reported no specific exposure location (Figure 2F).

### 3.3 Awareness and attitudes toward passive smoking

The data on awareness and attitudes toward passive smoking indicated a generally high level of correct knowledge and concern among participants (Table 1). Approximately 62.5% had heard of the term “passive smoking,” and 92.0% reported that they thought about it. A large majority (87.0%) recognized that smoke from cigarettes contributes to passive smoke, and 85.7% believed that shisha or water pipe smoke is harmful to non-smokers. Furthermore, 94.4% acknowledged that passive smoking can lead to health problems, and 95.0% were aware of the specific health issues it may cause. When asked who is most affected by passive smoking, 63.5% correctly identified the demographic, and 89.0% recognized that children and pregnant women are more vulnerable. Additionally, 93.7% agreed that children of smokers are more likely to develop respiratory problems, and 73.4% understood that babies of pregnant women exposed to passive smoke are at greater risk of congenital anomalies.

**Table 1:** Distribution of respondents according to awareness/attitude toward passive smoking.

**Awareness/Attitude for passive smoking**	**Incorrect Awareness/Attitude**		**Correct Awareness/Attitude**	
	**No**.	**%**	**No**.	**%**
Heard of the term “passive smoking”?	113	37.5%	188	62.5%
Think about passive smoking	24	8.0%	277	92.0%
Smoke from cigarettes contributes to passive smoke	39	13.0%	262	87.0%
Think smoke from shisha/water pipes is harmful to the non-smokers around	43	14.3%	258	85.7%
Think passive smoking can cause health problems	17	5.6%	284	94.4%
Health problems associated with passive smoking?	15	5.0%	286	95.0%
Demographic is most harmed by passive smoking-related diseases	110	36.5%	191	63.5%
Children and pregnant women are more vulnerable to the effects of passive smoking	33	11.0%	268	89.0%
Think that children of smokers are more likely to develop respiratory problems than children of non-smokers	19	6.3%	282	93.7%
Think that babies of pregnant women exposed to passive smoke have a higher chance of developing congenital anomalies	80	26.6%	221	73.4%
Think smoking should be banned in public places	43	14.3%	258	85.7%
Smokers should be encouraged to smoke in designated areas	35	11.6%	266	88.4%
It is acceptable for someone to smoke indoors if others are present	62	20.6%	239	79.4%
Non-smokers have the right to complain about passive smoking	74	24.6%	227	75.4%

Regarding attitudes, 85.7% believed that smoking should be banned in public places, and 88.4% supported encouraging smokers to use designated areas. Most participants (79.4%) disagreed with smoking indoors when others were present, and 75.4% agreed that non-smokers have the right to complain about exposure to passive smoke. These findings reflect a strong awareness of the dangers of passive smoking and a supportive attitude toward public health measures to reduce exposure.

### 3.4 Principal component analysis (PCA) of awareness and attitude items regarding passive smoking

The PCA of awareness and attitude items regarding passive smoking revealed a multidimensional structure, capturing underlying patterns in the participants' responses. Four principal components were extracted (Table 2).

**Table 2:** Principal component analysis of the awareness/attitude item.

**Awareness/Attitude Item**	**Principal component**			
	**Comp 1**	**Comp 2**	**Comp 3**	**Comp 4**
Heard of the term “passive smoking”?	0.336	0.548	−0.347	−0.404
Think about passive smoking	0.628	−0.133	0.045	−0.414
Smoke from cigarettes contributes to passive smoke	0.658	−0.077	0.1	−0.317
Think smoke from shisha/water pipes is harmful to the non–smokers around	0.637	−0.227	−0.009	0.08
Think passive smoking can cause health problems	0.659	−0.201	−0.171	0.212
Health problems associated with passive smoking?	0.563	−0.153	−0.262	0.451
Demographic is most harmed by passive smoking–related diseases	0.44	−0.1	0.113	−0.235
Children and pregnant women are more vulnerable to the effects of passive smoking	0.675	−0.096	0.028	−0.028
Think that children of smokers are more likely to develop respiratory problems than children of non–smokers	0.651	−0.063	−0.095	0.314
Think that babies of pregnant women exposed to passive smoke have a higher chance of developing congenital anomalies	0.485	0.43	−0.217	0.184
Think smoking should be banned in public places	0.077	0.423	0.438	0.329
Smokers should be encouraged to smoke in designated areas	0.415	0.087	0.528	−0.224
It is acceptable for someone to smoke indoors if others are present	0.15	−0.105	0.717	0.175
Non–smokers have the right to complain about passive smoking	0.269	0.752	0.098	0.113

Component 1 appeared to represent general awareness and understanding of passive smoking and its health effects, as it showed high positive loadings for items such as “Think passive smoking can cause health problems” (0.659), “Children and pregnant women are more vulnerable to the effects of passive smoking” (0.675), and “Smoke from cigarettes contributes to passive smoke” (0.658). This suggested that Component 1 reflects a broad knowledge base concerning the risks and populations affected by passive smoking.

Component 2 seemed to capture attitudinal endorsement and social responsibility, with the highest loading for “Non-smokers have the right to complain about passive smoking” (0.752) and “Heard of the term “passive smoking” (0.548). These items indicated a sense of individual and societal agency in addressing passive smoking issues.

Component 3 is characterized by items related to tolerance or acceptability of smoking behavior, notably “It is acceptable for someone to smoke indoors if others are present” (0.717) and “Smokers should be encouraged to smoke in designated areas” (0.528). These findings reflect varying degrees of acceptance or regulation of smoking practices in shared environments.

Component 4 included mixed loadings and may represent a residual or nuanced perception of health outcomes and policy. For example, “Health problems associated with passive smoking?” (0.451) and “Think smoking should be banned in public places” (0.329) load moderately, indicating specific concerns about consequences and regulation.

Overall, the PCA suggested that awareness and attitude items form coherent clusters representing knowledge, advocacy, behavioral tolerance, and policy perceptions, offering valuable insights for targeted interventions and educational efforts.

### 3.5 Overall awareness and attitude levels

The overall level of awareness and attitudes regarding passive smoking among participants was predominantly positive (Table 3). A substantial majority, comprising 85.7% (*n* = 258), demonstrated a good level of awareness and attitude, scoring above 66%. Moreover, 12.3% (*n* = 37) of the participants exhibited a fair level of awareness and attitudes, falling within the 33%-66% range. Only a small fraction, 2.0% (*n* = 6), showed a poor level of awareness and attitude, with scores below 33%. This distribution indicated a generally high level of understanding and appropriate attitudes toward the risks and implications of passive smoking among the surveyed population.

**Table 3:** Overall awareness/attitude level of passive smoking.

**Awareness/Attitude overall level about passive smoking**	**No**.	**%**
>66% (Good)	258	85.7%
33–66% (Fair)	37	12.3%
< 33% (Poor)	6	2.0%

### 3.6 Public perception and behavioral response to passive smoking

The data highlighted the extent to which individuals are affected by passive smoking and their attitudes toward it (Table 4). A majority of the participants reported encountering passive smoking in public places either occasionally (50.8%), often (18.3%), or very often (18.9%), whereas a smaller portion reported rarely (10.0%) or never (2.0%) smoking. A total of 84.7% of the respondents indicated that they always feel annoyed or uncomfortable when exposed to passive smoke, whereas 9.0% sometimes feel discomfort, and only 6.3% reported no discomfort. Regarding perceptions, 72.4% believed that passive smoking is a significant public health issue in the Hail region, with 19.6% being unsure and 8.0% disagreeing. Notably, there was overwhelming support (93.4%) for public awareness campaigns about the dangers of passive smoking. Furthermore, 68.8% of the participants stated that they had requested that someone stop smoking near them, reflecting proactive attitudes toward minimizing exposure.

**Table 4:** Distribution of the respondents based on the problems faced with passive smoking.

**Facing problems with passive smoking**		**No**.	**%**
Encounter passive smoking in public places	Never	6	2.0%
	Rarely	30	10.0%
	Occasionally	153	50.8%
	Often	55	18.3%
	Very often	57	18.9%
Feel annoyed or uncomfortable when exposed to passive smoking	No	19	6.3%
	Sometime	27	9.0%
	Always	255	84.7%
Think passive smoking is a significant public health issue in the Hail region	No	24	8.0%
	Do not know	59	19.6%
	Yes	218	72.4%
Support public awareness campaigns about the dangers of passive smoking	No	6	2.0%
	Not sure	14	4.7%
	Yes	281	93.4%
Ever requested someone to stop smoking near you	No	94	31.2%
	Yes	207	68.8%

### 3.7 Associations between awareness/attitude and sociodemographic variables

The associations between overall awareness and attitudes toward passive smoking and various sociodemographic variables revealed several significant findings (Table 5). Age showed a statistically significant association (*F* = 13.52, *p* < 0.001), with the highest awareness levels among individuals aged 18–30 years (mean = 85.75%, SD = 14.09), whereas those under 18 years and over 60 years had notably lower scores (58.24 and 42.86%, respectively). Gender also significantly differed (*t* = 3.54, *p* < 0.001), with females (mean = 86.71%) exhibiting higher awareness levels than males (mean = 80.06%).

**Table 5:** Association of overall awareness/attitudes with sociodemographic variables.

**Variable**	**Awareness/Attitude overall (%)**		**Significance**	
	**Mean**	**SD**	***F*****/*****t*** **value**	***p*** **value**
**Age**				
< 18 years	58.24	24.89	*F* = 13.52	**< 0.001**
18–30 years	85.75	14.09		
31–45 years	82.49	13.7		
46–60 years	81.2	20.92		
>60 years	42.86	50.51		
**Gender**				
Male	80.06	19.8	*T* = 3.54	**< 0.001**
Female	86.71	11.14		
**Educational level**				
Middle School or below	44.64	38.41	*F* = 15.97	**< 0.001**
High School	74.74	21.48		
College/University	85.87	12.81		
Postgraduate	83.52	19.43		
**Occupation**				
Education sector	84.42	14.99	*F* = 4.65	**< 0.001**
Government employee	77.14	23.1		
Healthcare Worker	86.84	12.66		
Medical Student	87.76	15.35		
Student (non–medical)	77.08	15.62		
Unemployed	81.62	14.24		
Military Sector	64.29	27		
Private/Self–Employed	71.43	21.93		
**Location**				
Urban	84.39	14.99	*F* = 4.36	**0.014**
Suburban	78.1	21.46		
Rural	74.79	24.89		
**Monthly income**				
Less than 5,000 SAR/month	84.86	14.59	*F* = 4.16	**0.016**
5,000–15,000 SAR/month	80	18.1		
More than 1,5000 SAR/month	77.09	24.33		
**Smoking Status**				
Smoker	73.89	23.86	5.79	**0.003**
Former smoker	80.45	11.87		
Non–smoker	84.5	15.55		

F/t value' refers to the test statistic obtained from either an ANOVA (F–test) or an independent samples t–test. Bold values indicate statistically significant differences at *p* < 0.05.

Educational level was strongly associated with awareness (*F* = 15.97, *p* < 0.001). The participants with a college/university education had the highest level of awareness (mean = 85.87%), whereas those with a middle school education or below had the lowest level of awareness (mean = 44.64%). Occupation also showed a significant relationship (*F* = 4.65, *p* < 0.001); medical students and healthcare workers had the highest awareness (87.76% and 86.84%, respectively), whereas individuals in the military and private/self-employed sectors had lower levels (64.29 and 71.43%, respectively).

Location significantly influenced awareness (*F* = 4.36, *p* = 0.014), with urban residents having the highest scores (mean = 84.39%) compared with suburban (78.10%) and rural residents (74.79%). Monthly income was also significant (*F* = 4.16, *p* = 0.016), with those earning less than 5000 SAR indicating the highest level of awareness (84.86%), whereas those earning more than 15000 SAR had comparatively lower scores (77.09%).

Finally, smoking status was significantly associated with awareness (*F* = 5.79, *p* = 0.003). Non-smokers had the highest awareness and attitude scores (mean = 84.50%), followed by former smokers (80.45%), whereas current smokers had the lowest awareness levels (73.89%). These findings suggest that younger, more educated, female, urban, and non-smoking individuals tend to have greater awareness and more positive attitudes toward the dangers of passive smoking.

### 3.8 Predictors of awareness and attitude: regression analysis

Univariate regression analysis was used to examine the relationships between awareness/attitude scores toward passive smoking and various demographic variables (Table 6). The intercept of the model was 49.51 (*p* = 0.001), indicating the baseline awareness/attitude score.

**Table 6:** Univariate regression analysis showing the relationships between the awareness/attitude scores and demographic variables.

**Parameter**	**B**	**SE**	***t*–value**	***p*–value**	**Effect size**
Intercept	49.51	14.47	3.42	**0.001**	0.04
< 18 years	7.67	11.81	0.65	0.517	0.002
18–30 years	24.16	11.24	2.15	**0.033**	0.016
31–45 years	24.38	11.29	2.16	**0.032**	0.016
46–60 years	29.26	11.64	2.51	**0.013**	0.022
> 60 years	0				
Male	−4.21	2.25	−1.87	0.063	0.012
Female	0				
Middle School or below	−28.83	9.28	−3.11	**0.002**	0.033
High School	−5.16	5.03	−1.03	0.306	0.004
College/University	−2.49	4.58	−0.54	0.587	0.001
Postgraduate	0				
Education sector	9.21	6.16	1.5	0.136	0.008
Government employee	3.18	6.75	0.47	0.639	0.001
Healthcare Worker	12.44	6.48	1.92	0.056	0.013
Medical Student	11.97	5.85	2.05	**0.042**	0.015
Student (non–medical)	3.28	6.47	0.51	0.612	0.001
Unemployed	4.47	6.07	0.74	0.462	0.002
Military Sector	0.84	7.86	0.11	0.915	0
Private/Self–Employed	0				
Urban	5.65	3.98	1.42	0.157	0.007
Suburban	1.77	4.62	0.38	0.702	0.001
Rural	0				
Less than 5,000 SAR/month	4.14	3.5	1.18	0.238	0.005
5,000–15,000 SAR/month	1.76	3.59	0.49	0.625	0.001
More than 15,000 SAR/month	0				
Smoker	−5.78	3.28	−1.76	0.08	0.011
Former smoker	−2.03	3.67	−0.55	0.582	0.001
Non–smoker	0				

“B” represents the unstandardized regression coefficient, indicating the expected change in awareness/attitude scores for each category compared with a reference group. “Effect size” quantifies the strength of the association between each predictor and the outcome variable, with higher values reflecting stronger relationships. Bold values indicate statistically significant differences at *p* < 0.05.

Age was a significant predictor, with individuals aged 18–30 years (*B* = 24.16, *p* = 0.033, effect size = 0.016), 31–45 years (*B* = 24.38, *p* = 0.032, effect size = 0.016), and 46–60 years (*B* = 29.26, *p* = 0.013, effect size = 0.022) having significantly higher awareness scores than those aged over 60 years (reference group). The group under 18 years of age was not significantly different (*p* = 0.517).

Gender was marginally associated with awareness scores, with males showing a nonsignificant negative association (*B* = −4.21, *p* = 0.063), suggesting a trend toward lower awareness than females did.

Education level played a key role: participants with only middle school education or below had significantly lower scores (*B* = −28.83, *p* = 0.002, effect size = 0.033), whereas those with high school or college/university education did not differ significantly from the postgraduate group.

Occupational status also influences awareness. Medical students (B = 11.97, p = 0.042, effect size = 0.015) had significantly higher scores, whereas healthcare workers showed a near-significant positive trend (*B* = 12.44, *p* = 0.056). Other occupations, including the education sector and unemployed individuals, did not show significant associations.

Location and monthly income were not significant predictors. However, urban residence showed a slight positive trend (*B* = 5.65, *p* = 0.157), and lower income levels (< 5,000 and 5,000–15,000 SAR/month) did not significantly influence awareness scores compared with higher income.

Smoking status revealed that current smokers had lower awareness scores (*B* = −5.78, *p* = 0.080, effect size = 0.011) than non-smokers did, indicating a possible negative association, although it was not statistically significant. There was no significant difference in the number of former smokers.

In summary, age, low educational attainment, and occupation, especially being a medical student, were significant predictors of awareness and attitudes toward passive smoking, whereas other factors, such as gender, location, and income, had limited or marginal influence.

## 4 Discussion

### 4.1 Sociodemographic profile and smoking status

The participants were predominantly young adults, which is consistent with global findings that this age group is a critical target for tobacco control because of both their vulnerability to exposure and their role in shaping future social norms around smoking (37, 38). The high educational level of the sample, including many medical students, likely contributed to more favorable awareness and attitudes toward passive smoking. Similar associations between higher education and greater health literacy have been reported in prior studies (39). The relatively low prevalence of current smokers in this group is encouraging, particularly in contrast with reports of higher smoking rates in comparable populations elsewhere (37, 38). However, since the majority of participants in our study were young and educated, the results mainly reflect a health-aware group and may not represent other populations with lower education levels or different socioeconomic backgrounds, where awareness and smoking habits could be very different.

### 4.2 Exposure to passive smoking

Despite a low proportion of active smokers, passive smoke exposure is common, especially in public spaces. This suggests that environmental and social contexts remain major contributors to SHS exposure, in line with findings from Indian and regional studies identifying public domains such as markets and transit hubs as hotspots for involuntary exposure (4). The persistence of household exposure, particularly from family members, underscores the need for interventions that extend beyond individual behavior to include community- and family-level strategies.

### 4.3 Awareness and attitudes toward passive smoking

Overall awareness of the health hazards of SHS was high, with most participants able to identify major risks and vulnerable groups. The disconnect between high awareness and prevalent exposure to SHS is perhaps the most significant finding warranting critical examination. However, as this was a cross-sectional study, the associations observed between awareness, attitudes, and exposure should be interpreted cautiously. While the findings highlight important patterns, the design does not allow causal inference, and it cannot be determined whether greater awareness leads to protective behaviors or if reduced exposure influences awareness levels. Although 84.1% of the participants were non-smokers and 94.4% recognized the health dangers of SHS, a substantial portion reported regular exposure, primarily in public spaces (60.8%) and at home. This paradox underscores a troubling gap between knowledge and policy enforcement. This finding suggests that awareness alone is insufficient to engender protective behaviors, particularly in the face of powerful social and cultural determinants. For example, the reluctance to confront a smoking family member at home or the ineffective enforcement of smoking bans in public areas can nullify individual knowledge. This aligns with studies in other contexts where knowledge does not automatically translate to action owing to ingrained social habits and weak regulatory oversight (40–44).

However, gaps persist, including limited familiarity with the term “passive smoking” and incomplete recognition of specific risks such as congenital anomalies. Similar knowledge gaps have been reported in other Indian studies, even among educated non-smokers (39, 45–47). Importantly, although most respondents supported bans and designated smoking zones, a minority still found indoor smoking acceptable, pointing to areas where social norms and enforcement require strengthening. This highlights the need for continuous education campaigns and stricter implementation of smoke-free policies (4, 48).

### 4.4 Principal component analysis of awareness and attitudes

The PCA provided deeper insights by revealing distinct clusters of knowledge and attitudes. One component reflected strong awareness of health risks and vulnerable groups, aligning with earlier studies linking health literacy to positive attitudes (4, 49). Another component highlighted advocacy and civic responsibility, indicating a willingness to translate awareness into proactive behaviors, similar to trends reported in Saudi Arabia and Nigeria (50, 51). In contrast, a third component revealed residual tolerance of smoking in shared spaces, reflecting gaps in policy enforcement and social acceptance, particularly among younger adults (4, 38). These findings emphasize the multidimensional nature of awareness and the importance of addressing not only knowledge but also behavioral norms and policy support.

### 4.5 Public perception and behavioral response

The participants' real-world experiences confirmed the continued prevalence of SHS exposure. A large proportion reported annoyance or discomfort when exposed, and most supported public awareness campaigns, suggesting growing public intolerance of SHS. These findings mirror global evidence of increasing social unacceptability of smoking in shared environments (50, 52, 53). Such attitudes can act as drivers for stronger enforcement of existing regulations and for the introduction of new smoke-free policies in high-exposure settings.

### 4.6 Sociodemographic associations

The analysis revealed that awareness was significantly influenced by age, education, and occupation. Younger participants and those with higher education consistently scored higher, confirming previous findings that these groups are more likely to engage with and internalize health information (24, 54). Medical students and healthcare workers also demonstrated particularly strong awareness, reflecting their educational training and professional orientation. Conversely, older adults, individuals with lower education, and smokers themselves had lower awareness levels, echoing patterns observed in other regional studies (36, 49, 51). These disparities highlight priority groups for targeted interventions, particularly older populations and those with less education.

### 4.7 Predictors of awareness and attitudes

Regression analysis confirmed that educational attainment and occupational status were the strongest predictors of awareness. Being a medical student significantly increased awareness, whereas lower education was associated with poorer knowledge. These findings emphasize the central role of education in shaping public health awareness. Although gender and income showed only marginal associations, the trends suggest that women and urban residents may have greater sensitivity to SHS risk, which is consistent with broader regional research (39, 50, 55, 56). Current smokers, while not significantly different in regression, demonstrated lower awareness scores, suggesting that denial or minimization of risks may influence their attitudes (49).

### 4.8 Implications for policy and practice

These findings underscore the critical need for multi-level interventions that address the persistent gap between high awareness of SHS risks and the adoption of protective behaviors. For example, although more than 70% of the respondents acknowledged the harms of SHS, fewer than half reported consistent preventive practices, such as maintaining smoke-free homes or avoiding exposure in public places. This discrepancy mirrors global surveys by the U.S. Centers for Disease Control and Prevention (1) and World Health Organization (2), which report rising awareness of SHS but inadequate compliance with smoke-free norms, particularly in developing regions (1, 2). This finding indicates that informational campaigns alone are insufficient and must be integrated with strategies that empower individuals and modify structural environments.

From a practical standpoint, public health campaigns should extend beyond information delivery to address psychosocial and cultural barriers. Efforts must enhance self-efficacy, especially among women and children, who may feel powerless in confronting smokers at home and promoting broader social norm changes. Stricter enforcement of smoke-free laws in public and semipublic spaces is also essential, with stronger monitoring, penalties, and community engagement to ensure compliance. Tailored strategies are needed for vulnerable subgroups, such as older adults, individuals with lower education or socioeconomic status, and smokers, who use community-based programs, peer education, and culturally sensitive approaches. Importantly, healthcare professionals and medical students, who already demonstrate greater awareness, should play proactive roles as advocates, counselors, and role models in tobacco control.

Theoretically, these findings highlight the value of integrating behavioral and environmental models in understanding SHS exposure. The health belief model explains why awareness does not always translate into action, pointing to the role of perceived barriers, cues to action, and self-efficacy (57). Social cognitive theory emphasizes how behaviors are reinforced in familial and social contexts where smoking may be normalized (58). The Environmental Health perspective broadens this lens, stressing the influence of policies, built environments, and socioeconomic structures (59). Together, these frameworks provide a nuanced understanding of SHS behavior in low- and middle-income settings, showing that individual knowledge must be supported by changes in social norms and structural conditions. This integrated approach suggests that future research should employ mixed methods to explore the cultural and relational dynamics that shape SHS exposure and avoidance, thereby informing more effective and holistic public health strategies.

## 5 Limitations and future directions

While the study offers valuable insights, certain limitations should be considered. First, the use of a convenience sampling method limits the generalizability of the findings to the broader population. Because no formal stratification or quota system was applied, the sample composition reflects natural variation from convenience recruitment, resulting in overrepresentation of urban (84.4%) and younger adults (65.1% aged 18–30 years). Second, the reliance on self-reported questionnaires may introduce response or social desirability bias, and the cross-sectional design captures associations only at a single point in time, without establishing causality. Third, the use of mixed-mode administration (online and paper-based), while necessary for accessibility, may have introduced mode effects, where differences between respondent groups (e.g., in digital literacy or age) could lead to measurement inconsistencies. Fourth, although trained research assistants were provided with standardized instructions, minor variability in the support they offered to participants cannot be entirely ruled out. Fifth, the sample showed age distribution bias, as a small number of adolescents (< 18 years) were included with parental consent, whereas older adults (>60 years) were underrepresented, limiting comparability across age groups. Finally, although the sample captured a range of educational and occupational backgrounds, the predominance of urban, highly educated participants may not reflect the perspectives of rural or less educated populations.

Future research should employ larger, more representative samples that include rural populations and a wider age spectrum. Longitudinal and interventional designs would allow assessment of changes over time and the impact of public health initiatives. Incorporating objective measures of exposure and exploring attitudes toward policy enforcement may further strengthen evidence to guide national tobacco control strategies.

## 6 Conclusion

In conclusion, this study highlights a high level of awareness and concern regarding the health risks of passive smoking among participants, along with strong support for smoke-free public policies. These insights underscore the need to reinforce educational initiatives and implement targeted regulatory measures aimed at reducing SHS exposure, especially in public spaces. By addressing key sociodemographic predictors of awareness and attitudes, such interventions can be more effectively tailored to high-risk populations, contributing meaningfully to national and global tobacco control efforts.

## Data Availability

The original contributions presented in the study are included in the article, further inquiries can be directed to the corresponding author.
